# A decision-analytical perspective on incorporating multiple outcomes in the production of clinical prediction models: defining a taxonomy of risk estimands

**DOI:** 10.1186/s12916-025-03978-3

**Published:** 2025-03-06

**Authors:** Glen P. Martin, Alexander Pate, Siân Bladon, Matthew Sperrin, Richard D. Riley

**Affiliations:** 1 Division of Informatics, Imaging and Data Science, Faculty of Biology, Medicine and Health, University of Manchester, Manchester, UK; 2 Institute of Applied Health Research, College of Medical and Dental Sciences, University of Birmingham, Birmingham, UK

**Keywords:** Prediction modelling, Estimand, Multiple outcome, Multivariate, Multi-label

## Abstract

**Background:**

Clinical prediction models (CPMs) estimate an individual’s risk of current or future outcome events, using information available about the individual at the time of prediction. While most CPMs are developed to predict a single outcome event, many clinical decisions require considering the risks of multiple outcome events. For example, decision-making for anticoagulation therapy involves assessing an individual’s risks of both blood clot and bleeding, while decision-making around interventions for multimorbidity prevention requires an understanding of the risks of developing multiple long-term conditions. However, determining when and how to incorporate multiple outcomes into CPMs remains challenging. This article aims to raise awareness of multiple outcome prediction and present clinical examples where such prediction is essential to help inform individual decision-making.

**Main text:**

A range of analytical methods are available to develop multiple-outcome CPMs, but there are frequent malapropisms and heterogeneity in terminology across this literature, making it difficult to identify/compare possible methods. Selecting the appropriate method should depend on the intended risk estimand—the type of predicted risks that we wish the CPM to estimate—but this is often not defined or reported. Using clinical examples and a decision-analytical perspective, we present a taxonomy of risk estimands to frame different clinical contexts requiring multiple-outcome CPMs. We outline four levels of risk estimands: (i) single-outcome risk, (ii) competing-outcome risk, (iii) composite-outcome risk, and (iv) risk of multiple outcome combinations. We demonstrate how a decision-analytical and utility-theory lens can help define the risk estimand for a given clinical scenario, based on the model’s intended use.

**Conclusions:**

Clearly defining and reporting the risk estimand is essential for all prediction model studies. A decision-analytical framework aids in selecting the most appropriate estimand for a given prediction task and in determining when and how to incorporate multiple outcomes into CPM development.

## Background

Clinical prediction models (CPMs) are multivariable regression models or machine learning algorithms that estimate an individual's risk of an outcome event using their observed characteristics [1, 2]. Examples include QRisk, which estimates an individual’s risk of developing cardiovascular disease to support decision-making around lifestyle/ medication interventions [3], and PLCO_M2012_, which predicts the risk of lung cancer to support decision-making in lung-health checks [4].

Most CPMs focus on predicting a single outcome event (label). This can be insufficient when a holistic assessment of a patient’s health is needed. For example, in primary prevention of disease, one needs to consider the risks of different outcome events that might occur for an individual throughout their life and how they interact. It can also be insufficient when CPMs are used to support individual decision-making where end-users need to consider multiple possible events and their patient-specific utilities. For instance, decision-making for anticoagulation therapy requires balancing an individual’s risks of blood clot (if untreated) against the risk of bleeding (if treated), taking into account their preferences and the utility of treatments and outcome events. These outcomes are not mutually exclusive (nor independent), so the methods to develop a CPM to support such decision-making needs to account for such multiple outcomes.

There is increasing methodological advancement around using multivariate (defined throughout as multiple outcomes) modelling methods for developing and validating CPMs [5–13]. However, frequent malapropisms (incorrect use of “multivariate” to mean multiple covariates) and heterogeneity (interchangeable use of “multivariate”, “multi-label”, “multi-output” and “multi-target”) in the terminology makes accessing the literature difficult [14]. Moreover, the methods for incorporating multiple outcome events into CPMs depend on the types of predicted risks, or risk estimand, that the CPM targets but this is often not reported making it difficult to identify/compare possible methods [15].

To increase understanding of when, and how, to incorporate multiple outcome events into CPMs for a broad audience, we outline a taxonomy of risk estimands that a multivariate CPM might target and suggest methods to develop such CPMs. To motivate this, we present, using decision-analysis and utility theory [16], clinical examples where incorporating multiple outcome events into a CPM is needed to inform individual decision-making. Hereto, we use the term ‘outcomes’ to mean ‘outcome events’, for brevity.

## Example of single outcome prediction and decision-analytical methods

To begin, imagine we wish to develop a diagnostic CPM to help decision-making around referrals to CT scans for a definitive diagnosis of lung cancer. Defining the outcome as being whether someone has lung cancer or not, we might then use logistic regression, or a suitable machine learning model, to develop a CPM for estimating if an individual has lung cancer. Assuming the necessary model validation steps show this model is well calibrated and has clinical utility [1, 2], we might then use it to help decide whether to undertake a CT scan for an individual. Decision-analytical frameworks [16] formalise this idea.

**Fig. 1:**
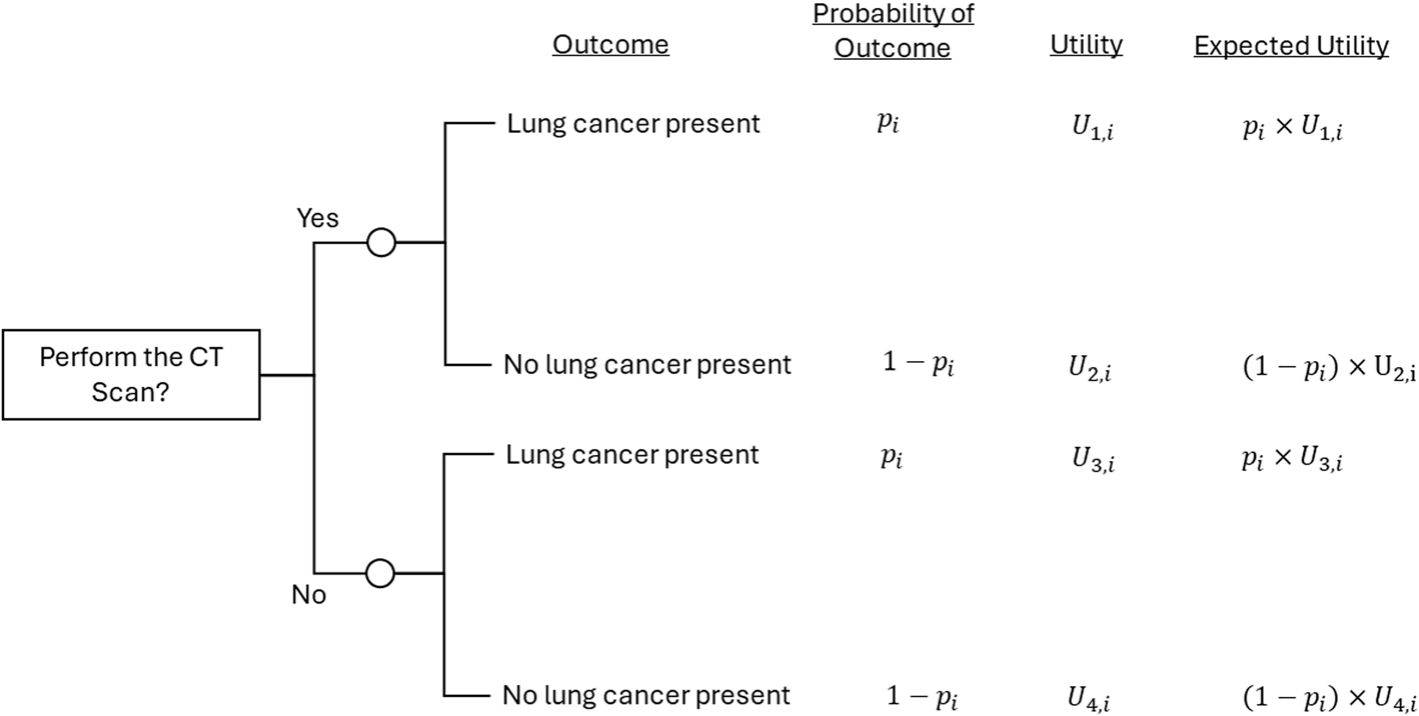
Decision-analytical diagram for whether to perform a CT scan for diagnosis of lung cancer. ${p}_{i}$ denotes an individual $i$’s risk of having lung cancer, as estimated from a well-calibrated prediction model, where ${U}_{1,i}, {U}_{2,i}, {U}_{3,i}$ and ${U}_{4,i}$ denote a given individual $i$’s utilities of each pathway

Decision-analytical methods weigh the relative merits of possible outcomes of a binary decision, based on the probability of them occurring and their utilities. In our example, we are deciding whether to perform a CT scan and there are two possible outcomes, creating four pathways (Fig. 1). For each pathway, a utility captures the relative benefits (e.g. confirmed diagnosis of lung cancer and initiation of treatment) and harms (e.g. radiation exposure) of taking the action, conditional on the outcome event. The utilities are person-specific and can include personal preferences. The individual’s utilities then imply the optimal risk threshold from the CPM at which the CT scan should be performed [16]. For the purpose of this paper, we assume that the person-specific utilities are available; however, these can be challenging to elicit [17].

Imagine our hypothetical CPM gives an individual’s predicted risk of lung cancer (denoted ${p}_{i}$) as 10%. The individual defines their utility of doing the CT scan when they have lung cancer (denoted ${U}_{1,i}$) to be 10 times more valuable than doing the CT scan when they do not have lung cancer (${U}_{2,i}$), and 5 times more valuable than not doing the CT scan when they do not have lung cancer (${U}_{4,i}$); not doing the scan when they do have lung cancer (${U}_{3,i}$) has no utility. For example, set ${U}_{1,i}=50, {U}_{2,i}=5, {U}_{3,i}=0$ and ${U}_{4,i}=10$. Then, the expected utility of doing the CT scan is $\left({p}_{i} \times {U}_{1,i}\right)+\left(\left(1-{p}_{i} \right)\times {U}_{2,i}\right)=9.5$, and the expected utility of not doing the CT scan is $\left({p}_{i} \times {U}_{3,i}\right)+\left(\left(1-{p}_{i} \right)\times {U}_{4,i}\right)=9$, suggesting the CT scan should be performed. Viewing prediction problems through a decision-analytical lens focusses on consideration of what the risk estimand is for a given clinical scenario, driven by how the model will be used.

## Examples of multiple outcome prediction

We now use three additional examples to illustrate common reasons to incorporate multiple outcomes into the CPM development. Throughout all examples, we define the risk estimands and probabilities of outcomes as being ‘in absence of the intervention’ (see the “Conclusion” section for a note on causal/counterfactual prediction under interventions). This is the natural choice since the utility of intervention is often related to (for example) a relative risk reduction in the outcome probability.

### Example 1: prediction of cardiovascular risk

**Fig. 2:**
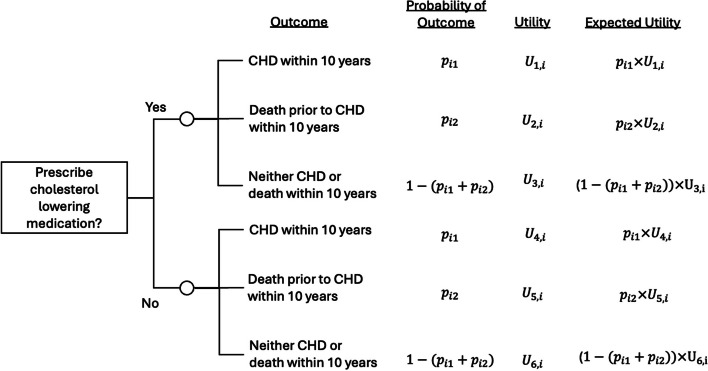
Decision-analytical diagram for whether to prescribe cholesterol-lowering medication for coronary heart disease (CHD) risk. ${p}_{i1}$ denotes an individual $i$’s risk of having CHD in the next 10 years, ${p}_{i2}$ is an individual’s risk of non-CHD death within the next 10 years, both as estimated from a well-calibrated prediction model accounting for the competing risks. Here, ${U}_{1,i}\text{ to }{U}_{6,i}$ denote a given individual $i$’s utilities of each pathway

Imagine we wish to develop a CPM to estimate an individual’s risk of developing coronary heart disease (CHD) within the next 10 years, to help inform the prescription of cholesterol-lowering medication. The decision-analytical diagram is shown in Fig. 2, illustrating that we need to estimate an individual’s risk of CHD in the next 10 years accounting for death from other causes [11]. An individual might consider the risks of the competing events in their decision-making of whether to take new medications (e.g. quality of life considerations). That is, the CPM should estimate the risk of multiple outcomes: CHD within 10 years and death due to non-CHD causes within 10 years, all without taking the medication. The individual predicted risks, combined with an individual’s utilities, then help inform the decision. For example, the decision-analytical diagram (Fig. 2) shows that the expected utility of prescribing the medication is $\left({p}_{i1} \times {U}_{1,i}\right)+\left({p}_{i2} \times {U}_{2,i}\right)+\left(\left(1-\left({p}_{i1}+{p}_{i2}\right)\right)\times {U}_{3,i}\right)$ and similarly for the expected utility of not prescribing the medication.

### Example 2: prediction of clinical deterioration in COVID-19

**Fig. 3:**
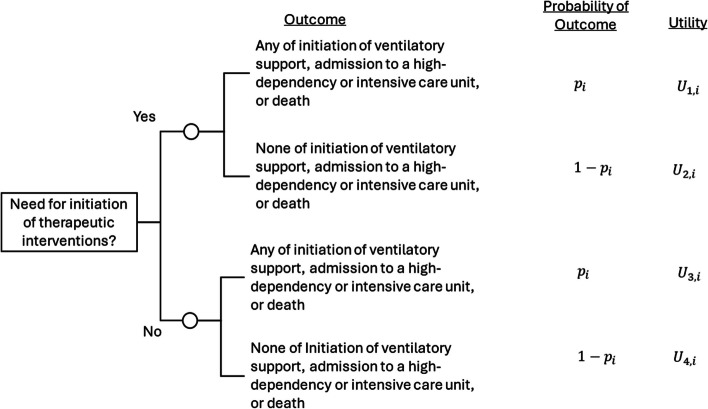
Decision-analytical diagram for interventions to lower risk of clinical deterioration in patients with COVID-19 who are admitted to the hospital. The decision-making is such that one needs an overall assessment of deterioration risk, so the outcome is a composite of multiple outcomes: initiation of ventilatory support, admission to a high-dependency or intensive care unit, or death. ${p}_{i}$ denotes an individual $i$’s risk of having any of the outcome events, as estimated from a well-calibrated prediction model. ${U}_{1,i} \text{to }{U}_{4,i}$ denote a given individual $i$’s utilities of each pathway

Imagine we wish to develop a CPM to predict an individual’s risk of clinical deterioration if admitted to a hospital with COVID-19 to help inform the need for therapeutic interventions. Here, we might not need to differentiate between the specific type of clinical deterioration to help make this decision, so we might define clinical deterioration as any of the following events: initiation of ventilatory support, admission to an intensive care unit, or death (e.g. Gupta et al. [18]). The decision-analytical diagram for this example is given in Fig. 3. The key difference compared to Fig. 1, is that the outcome is now defined as a composite of multiple events.

### Example 3: prediction combinations of ischaemic stroke and major bleeding

**Fig. 4:**
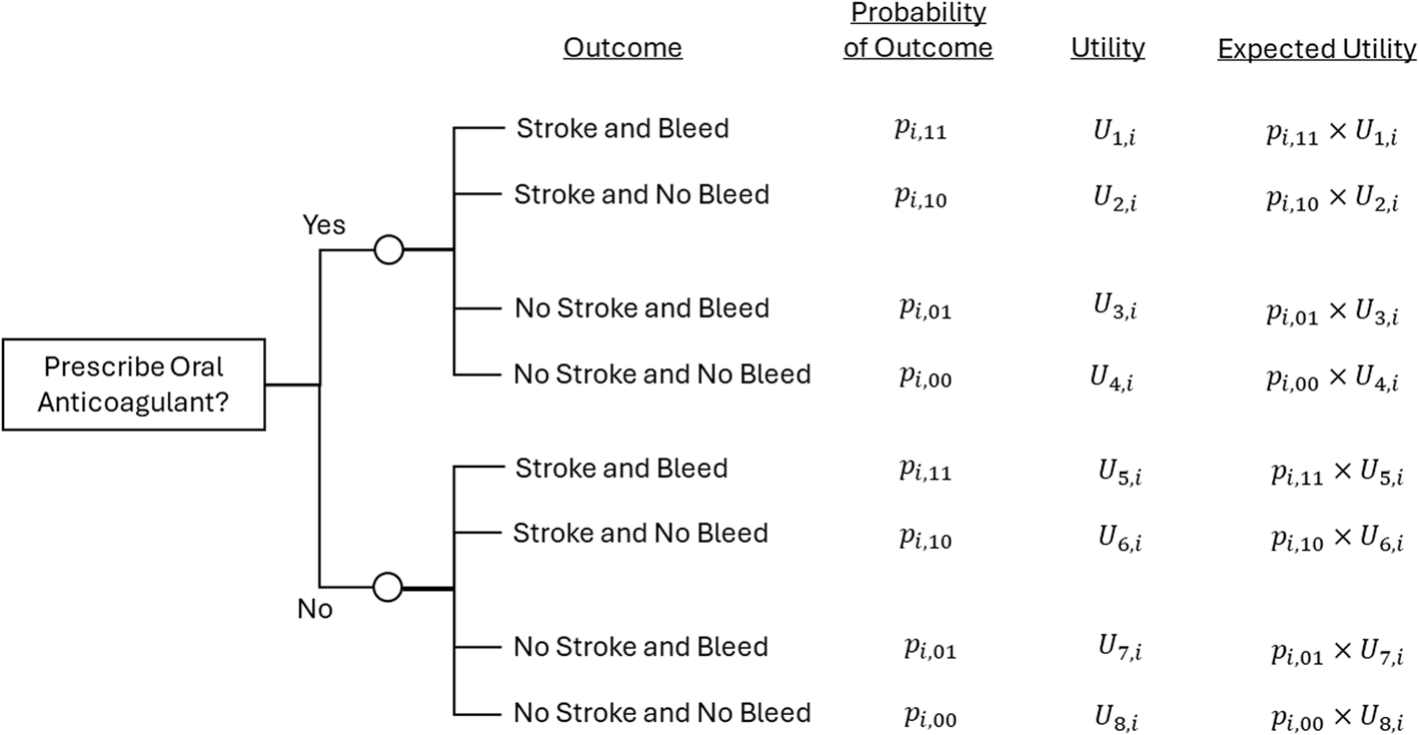
Decision-analytical diagram for prescribing oral anticoagulants based on individual prediction of both benefits and harms. ${p}_{i,11}$, ${p}_{i,10}$, ${p}_{i,01}$ and ${p}_{i,00}$ define an individual $i$’s risk of having ischaemic stroke and major bleed, ischemic stroke and no major bleed, no ischemic stroke and major bleed, and no ischemic stroke and no major bleed, respectively, as estimated from a prediction model (such that ${p}_{i,11}+{p}_{i,10}+{p}_{i,01}+{p}_{i,00}=1$). ${U}_{1,i} \text{to}{U}_{8,i}$ denote a given individual $i$’s utilities of each pathway

Imagine we wish to develop a CPM to help inform the prescription of anticoagulants in patients with atrial fibrillation accounting for the risks of two outcomes: ischaemic stroke and major bleeding. We need to consider the expected reduction in ischaemic stroke risk, traded-off against that individual’s major bleeding risk. We could develop a CPM that estimates ischaemic stroke risk and proceed like Fig. 1, capturing the harms (bleeding risk) within the utilities of each pathway. However, that does not allow the risk of those harms to vary based on the individual patient’s characteristics—it assumes that they are fixed. Therefore, we might extend the decision-analytical diagram as in Fig. 4. The risk estimand is now an individual’s risk of developing different combinations of ischaemic stroke and major bleeding, without taking an anticoagulant [19], where multivariate methods are needed (see “Taxonomy level 4: risk estimand is the predicted risk of different combinations of multiple outcomes” section). One would combine these risk estimates with the corresponding utilities to calculate the expected utility of prescribing and not prescribing oral anticoagulants (Fig. 4).

## Taxonomy of risk estimands for multiple outcome prediction

We now frame a taxonomy of risk estimands that one can estimate from a CPM (Table 1).

**Table 1:** Summary of taxonomy of risk estimands that one can estimate from a clinical prediction model (CPM), with references to example methods that can develop CPMs for each estimand. These considerations should be made alongside all other CPM design considerations (e.g. prediction horizon, outcome definition, predictor definitions, and so forth, as described by previous guidance resources [ 1, 2, 20 ])

**Taxonomy level 1: risk estimand is the predicted risk of a single outcome** This estimand is appropriate in clinical situations where actions based on the CPM only depend on a single outcome of interest, and where that outcome cannot be prevented from being observed by other outcomes. The context must be such that other outcomes don’t factor into the decision-analytical processes (“Example of single outcome prediction and decision-analytical methods” section). This estimand is what is targeted by most CPMs. It might be possible to utilise correlation between multiple outcomes to help improve the predictive performance of one of the outcomes using either multivariate modelling technique (e.g. [6, 8, 9]) or machine learning approaches (e.g. [5, 9, 21]).
**Taxonomy level 2: risk estimand is the predicted risk of multiple competing outcome events** This estimand is appropriate in clinical situations where interest is either solely on the risk of a single outcome that can be prevented by other outcomes, or on the risk of multiple competing events (example 1). In either case, competing risk methodologies are required [10, 11], to ensure the risks are estimated in a real-world that allows for the competing events to happen.
**Taxonomy level 3: risk estimand is the predicted risk of a composite of multiple outcomes** This estimand covers situations where one combines multiple outcomes into a new single composite outcome, and a univariate (single outcome) CPM is developed. This is only appropriate in clinical situations that do not need the CPM to help in differentiating between the different outcomes (example 2)—i.e. where clinical decisions are the same regardless of which of the multiple outcome events occurs. We recommend caution in developing CPMs for this estimand.
**Taxonomy level 4: risk estimand is the predicted risk of different combinations of multiple outcomes** This estimand is appropriate in situations where the risk of each bespoke combination of multiple outcome events happening is needed to inform decision-making (example 3). This requires the CPM to be fitted using multivariate modelling techniques that directly estimate, or account for, the correlation between the multiple outcomes (e.g. see Martin et al. for multiple binary outcomes [6], and Pate et al. [8] or Hougaard [13] for multiple time-to-event outcomes). Depending on the exact methods used, and type of outcome, such methods can also estimate the other risk estimands in the taxonomy from the same model.

### Taxonomy level 1: risk estimand is the predicted risk of a single outcome

Actions based on the CPM might only depend on a single outcome of interest. Here, the risk estimand is the probability of that outcome, conditional on the set of predictor variables. For the example in Section "Example of single outcome prediction and decision-analytical methods", the risk estimand is an individual’s diagnostic risk of having lung cancer at time of prediction, conditional on their predictor variables. We write this as $P(\text{lung cancer present}=1|X)$, where $X$ is the set of predictor variables (e.g. age and sex). In defining risk estimands for predicting a single outcome, one needs to ensure that the outcome cannot be prevented by other outcomes (see “Taxonomy level 2: risk estimand is the predicted risk of multiple competing outcome events” section).

The simplest way to develop a model for this risk estimand is to fit a univariate (single outcome) model for the outcome of interest. For example, using methods like logistic regression or random forest models for a binary outcome or an appropriate survival analysis model for a time-to-event outcome [1, 2]. This is how the majority of CPMs are developed.

There may be other outcomes in the development dataset that can be utilised to improve the prediction of the main outcome of interest. For example, Heider et al. (2013) [22] developed a CPM to predict an individual’s risk of being resistant to different antiretroviral treatments for human immunodeficiency virus, using mutation information as predictors. The model was developed using methods that utilise correlations between the different outcomes (resistance to different antiretroviral treatments) to improve the prediction of each outcome in turn (i.e. mutations leading to resistance against one drug might also lead to resistance against another drug) [23]. Utilising correlations like this to develop a CPM might be particularly helpful when some patients have missing outcome data, and we could “borrow information” from correlated outcomes (akin to ‘borrowing strength’ in multivariate meta-analysis [24]). Here, one could make use of multivariate modelling methods (“Taxonomy level 4: risk estimand is the predicted risk of different combinations of multiple outcomes” section), or “multi-label” machine learning approaches [5, 9, 21, 23].

### Taxonomy level 2: risk estimand is the predicted risk of multiple competing outcome events

The clinical context might be such that the main outcome can be prevented from occurring by other events. Our example 1 illustrates this. Here, we need to estimate the risk of the single outcome event of interest in a real world where individual can experience the competing events. This will mean developing the CPM using competing risk analysis methods; see Putter et al. [11] and van Geloven et al. [25]. Such models will output the risk of each competing event in turn, which may also be of interest to the decision-analytical framework (as in example 1).

### Taxonomy level 3: risk estimand is the predicted risk of a composite of multiple outcomes

If interest is in predicting if *any* of multiple outcomes occur for an individual, then we can define our risk estimand as the risk of the composite outcome. Example 2 illustrates this, where we would write the risk estimand as $P(\text{mechanical ventilation}\cup \text{intensive care unit}\cup \text{death}=1|X)$, where ‘$\cup$’ (union) means “or”. One converts the multiple separate outcomes into a single binary outcome defined as occurring if a patient has any of the individual outcomes. A univariate CPM (see “Taxonomy level 1: risk estimand is the predicted risk of a single outcome” section) can then be fit to this composite outcome.

This risk estimand is only appropriate when end-users do not need to distinguish between the individual components. Composite outcomes can be useful in situations where the sample size available for model development is limited [26]. For example, if any of the individual outcomes are rare, then creating a composite endpoint will increase outcome prevalence, in turn decreasing the required sample size. However, composite outcomes result in a loss of information, can increase heterogeneity in outcome definitions across studies and can present interpretation challenges [27]. Therefore, we recommend caution in developing CPMs for this estimand.

### Taxonomy level 4: risk estimand is the predicted risk of different combinations of multiple outcomes

Actions based on the CPM might depend on the risks of each bespoke combination of multiple outcomes co-occurring. This is illustrated in example 3, where a primary focus would be the risk of avoiding both ischaemic stroke and major bleeding to help inform medication prescription; we would write this as $P(\text{stroke}\cap \text{bleed}=0|X)$, where $\cap$ (joint) means “and”. This risk estimand could also be called the “joint risk of multiple outcomes being modelled”. Estimation of joint risk requires the use of methods that directly model, or account for, the correlation between the multiple outcomes being modelled. Developing a model for each outcome individually and then multiplying the resulting risks is inappropriate [6, 8].

One approach is to define a new outcome as being the combination of the multiple outcomes that one is interested in predicting [8]. In example 3, we might define a new binary variable as occurring if an individual experiences both an ischaemic stroke and a major bleed. One could then fit a univariate CPM (Section 0) to this new ‘joint’ outcome event. Whilst this is a simple approach and can perform well [8], it does not allow prediction of different outcome combinations.

To overcome this, one could define each combination of the multiple outcomes as a nominal outcome and use multinomial logistic regression to fit the CPM [6]. This model can then estimate an individual’s risk of each combination of the multiple outcomes of interest. Alternative approaches to estimating joint risk include—but not limited to—multivariate prohibit models [6], copula methods [28] and latent variable approaches [29]. See Pate et al. [8] and Hougaard [13] for potential methods to estimate the joint risk of multiple time-to-event outcomes. An advantage of using such techniques is that they produce a CPM that can also estimate the other risk estimands covered in our taxonomy from the same model.

## Special cases

### Nominal outcomes

Hereto, we have focussed on developing CPMs where there are multiple different outcomes. Sometimes, there might be a single outcome that itself has multiple ‘levels’ that are mutually exclusive (i.e. nominal/multi-class outcomes). For example, prediction of expected response to rheumatoid arthritis medication [30] (non-response, discontinuation due to adverse reactions, and response). Multinomial logistic regression is an appealing modelling approach [31]. We see this type of predicted risk sitting within taxonomy level 1 given that the focus is on predicting the single (nominal) outcome event. The decision-analytical diagram would be like Fig. 1 except that there would be more pathways, one per nominal outcome level, where clinical actions might differ across the outcome levels.

### Conditional risk in a multistate survival context

A special-case in a time-to-event context is where there is interest in exploring how the risk of one main time-to-event changes conditional on experiencing certain intermediate events through time. Multi-state models [11, 12] provide a natural modelling framework for this type of predicted risk, although there are alternative (simulation-based) methods. For example, Owen et al. [32] developed a multi-state model considering the temporal ordering of individuals developing psychosis, diabetes, congestive heart failure and/or death. The decision-analytical process compared an individual's predicted life expectancy without any disease diagnosis to their risk after one or more diagnoses through time. For such clinical question, we might target any of the risk estimands outlined in Table 1. For example, we might aim to estimate the time until diabetes and cardiovascular disease co-occur (taxonomy level 3) conditional on intermediate events (e.g. before/after being identified as pre-diabetic) [8]. Alternatively, as in Owen et al. [32], we could estimate the risk of a single outcome of interest (e.g. death; Taxonomy Level 1) conditional on intermediate events (e.g. before/after diagnosis of chronic conditions).

## Conclusion

We have framed the handling of multiple outcomes in prediction modelling within a taxonomy of risk estimands. Incorporating multiple outcomes into CPMs is gaining popularity, given many medical contexts in which such models have value, but the type of predicted risks that are targeted (risk estimand) is rarely stated. Failing to clearly define the risk estimand makes it difficult to compare methods, and creates challenges for researchers to decide why, when, and how to incorporate multiple outcomes into the CPM production.

Incorporating multiple outcomes into the development and validation of CPMs is an active area of research. The validation of the predictive performance of CPMs for joint risk prediction requires additional considerations [7], and there remains scope for novel methods of assessing calibration. Additionally, the examples in this paper involve multiple outcomes of the same ‘type’. Sometimes, the multiple outcomes might be of mixed ‘type’ (e.g. mixture of continuous, binary and time-to-event outcomes). Statistical methods for modelling mixed outcome ‘types’ have been proposed [33–35], but their application to the development of CPMs is rare, and the benefits of doing so remain uncertain. Finally, sometimes a CPM is used to make decisions for future prognosis where doing the action might alter the probability of each outcome. In such cases, the CPMs may need to consider causal effects [15, 36]. Whist we avoided such considerations for simplicity, all the decision-analytical diagrams considered could have been expanded with more branches to capture what treatment did (e.g. an individual counterfactually would have had stroke if untreated but the treatment avoided stroke); future research could explore methods for counterfactual prediction of multiple outcome events. Similarly, in practice the decision-analytical diagrams might be more complex than those presented here, with a larger set of clinical decisions that need to be made for an individual (e.g. patients with diabetes switching to new therapy/treatment through time); the concepts and principles covered still apply to such situations, and multi-criteria decision-making methods may be particularly useful in such situations [37].

An important consideration with any prediction modelling study is obtaining high-quality outcome and predictor data [1]. We note that one challenge with multiple-outcome prediction is the requirement to obtain appropriate high-quality data on the multiple outcomes. Electronic health records, which contain a holistic view of a patient’s medical diagnoses, may help with this [38].

In conclusion, it is recognized that one needs an unambiguous definition of the outcome when planning the development of CPMs [1]. We argue that the risk estimand of all CPMs should be defined and reported with the same rigour. Framing prediction problems within a decision-analytical framework can help identify the most appropriate risk estimand for a given prediction task, as well as illustrating when (and, in turn, how) to incorporate multiple outcomes into CPM production.

## Data Availability

No datasets were generated or analysed during the current study.

## Abbreviations

CHD: Coronary heart disease
CPMs: Clinical prediction models
